# Standardizing Anaphylaxis Treatment in Pediatric Care Settings

**DOI:** 10.1097/pq9.0000000000000652

**Published:** 2023-05-22

**Authors:** Sara Anvari, Vibha Szafron, Tanya J. Hilliard, Lisa Forbes-Satter, Mona D. Shah

**Affiliations:** From the * Department of Pediatrics, Texas Children’s Hospital, Section of Immunology, Allergy and Retrovirology, Baylor College of Medicine, Houston, Tex.; † Department of Pediatrics, Texas Children’s Hospital, Section of Hematology/Oncology, Baylor College of Medicine, Houston, Tex.

## INTRODUCTION

Anaphylaxis is a severe, rapid-onset hypersensitivity reaction with multisystem organ involvement.^1^ The reported lifetime prevalence of anaphylaxis is estimated to range from 1.6% to 5.1%.^2,3^ Despite established medical guidelines,^1,2,4^ misconceptions regarding the recognition and treatment of anaphylaxis continue to persist among healthcare providers, patients, and their caregivers, leading to delays in care and inadequate treatment.^5^

The most common cause of anaphylaxis in children and adults includes food, medication, and venom hypersensitivity.^6^ Approximately 20% of anaphylaxis-related fatalities are due to medications.^5^ Delayed or inappropriate treatment of anaphylaxis can be fatal. Intramuscular (IM) epinephrine is the first-line treatment for the management of anaphylaxis.^4^ IM epinephrine [administered at 0.01 mg/kg of a 1:1,000 concentration (maximum dose: 0.5 mg in adults and 0.3 mg in children)] be administered in the mid-anterolateral thigh is recommended for any episode of anaphylaxis.^4^ Antihistamines have a slow onset of action and are never used as the first-line treatment of anaphylaxis.^2,7^ There is limited evidence regarding the clinical benefit of glucocorticoids, which should also be avoided in the first-line treatment of anaphylaxis.^2,8^

Shaker et al^2^ describe the diagnosis of anaphylaxis based on clinical criteria (Table 1). Prompt assessment and early recognition of the signs and symptoms of anaphylaxis will ensure accurate diagnosis and timely administration of epinephrine, which can be life-saving by preventing progression to a fatal reaction.

**Table 1.** Anaphylaxis Criteria

Anaphylaxis Criteria	Exposure and Timing of Symptom Onset	Symptom Requirement
Criteria 1	Symptoms within minutes to hours after any exposure	Skin and/or mucosal tissue involvement and one of the following: a) respiratory compromise (eg, dyspnea, wheeze-bronchospasm, stridor, reduced peak flow, and hypoxemia); and b) reduced blood pressure or associated symptoms of end-organ dysfunction (eg, collapse, syncope, and incontinence)
Criteria 2	Symptoms within minutes to hours after exposure to likely allergen exposure	Two or more of the following: a) involvement of the skin-mucosal tissue; b) respiratory compromise; c) reduced blood pressure or associated symptoms; and d) persistent gastrointestinal symptoms (eg, abdominal pain and vomiting)
Criteria 3	Symptoms within minutes to hours after exposure to Known allergen	Reduced blood pressure

Anaphylaxis criteria as described in Shaker 2020 Anaphylaxis Criteria.^4^

Anaphylaxis has been reported with the use of biologics and chemotherapeutic agents.^9,10^ Because third-party payers may deny an inpatient admission for these therapies, clinicians often administer them in the outpatient setting.

Two unique cases of anaphylaxis led to the creation of the Anaphylaxis Work Group (AWG) at our center. Both cases took place in our outpatient infusion center. Case 1 involved a pediatric patient who experienced difficulty breathing and urticaria during a chemotherapy infusion with an agent known to cause anaphylaxis). The staff initially administered diphenhydramine, but symptoms persisted. At the time, the monitoring staff were unclear about whether to administer intravenous (IV) or IM epinephrine to treat anaphylaxis. In addition, when retrieving the epinephrine from the Omnicell (Omnicell, Santa Clara, Calif.), the appropriate needle gauge required for medication administration was unavailable. This issue led to further delays in emergent care. Ultimately, the staff administered IM Epinephrine, and the patient recovered without further complications.

Case 2 involved a pediatric patient who experienced symptoms of cough and rash during a chemotherapy infusion. The staff identified this as a case of anaphylaxis, but they administered an inadequate dose of IV epinephrine. Persistent symptoms led to transfer to the intensive care unit, where the patient required a continuous epinephrine infusion but recovered without further sequelae.

These events led to a formal evaluation to improve our center’s recognition and management of anaphylaxis. In this report, we describe our methodology and propose quality improvement tools for monitoring the impact of our interventions.

## METHODS

To review the events that led to the delay in treatment for case 1 and the inappropriate dose of IV epinephrine in case 2, the nurse leader and physician director of the outpatient infusion center scheduled a debriefing meeting with the infusion center staff members within 24 hours of each event. During each debrief, the leaders facilitated open-ended discussions with the team members. In debriefing these episodes, the treating team identified significant variations within the staff regarding their understanding and recognition of anaphylaxis and its prescribed first-line intervention. This discussion generated a multidisciplinary assessment team, with physician, nursing, and pharmacy representation from across our integrated delivery system (ie, a quaternary care center with multiple hospitals and outpatient centers), the AWG. Here, we describe the aims of the AWG, which included: (1)identification of areas of weakness regarding anaphylaxis to improve and monitor progress; (2) improved staff recognition and confidence in treating anaphylaxis with epinephrine based on World Allergy Organization (WAO) guidelines^1^; (3) accurate and easily accessible Electronic Medical Records (EMR) order set (Epic Systems Corporation, Verona, Wis.) and algorithm to manage and outpatient anaphylaxis episode; and (4) ability to assess the efficacy and safety of order set and algorithm through survey response. In the event of an anaphylactic reaction, the patient would be referred to 1 of our 5 Allergy and Immunology (A/I) clinics for evaluation and management of anaphylaxis. Across all sites, our A/I clinics see approximately 16,000 A/I patients annually, with 800 patients managed for drug-induced anaphylaxis. Our A/I clinics are staffed with 18 clinical nurses and 22 medical providers.

**Fig. 1.**
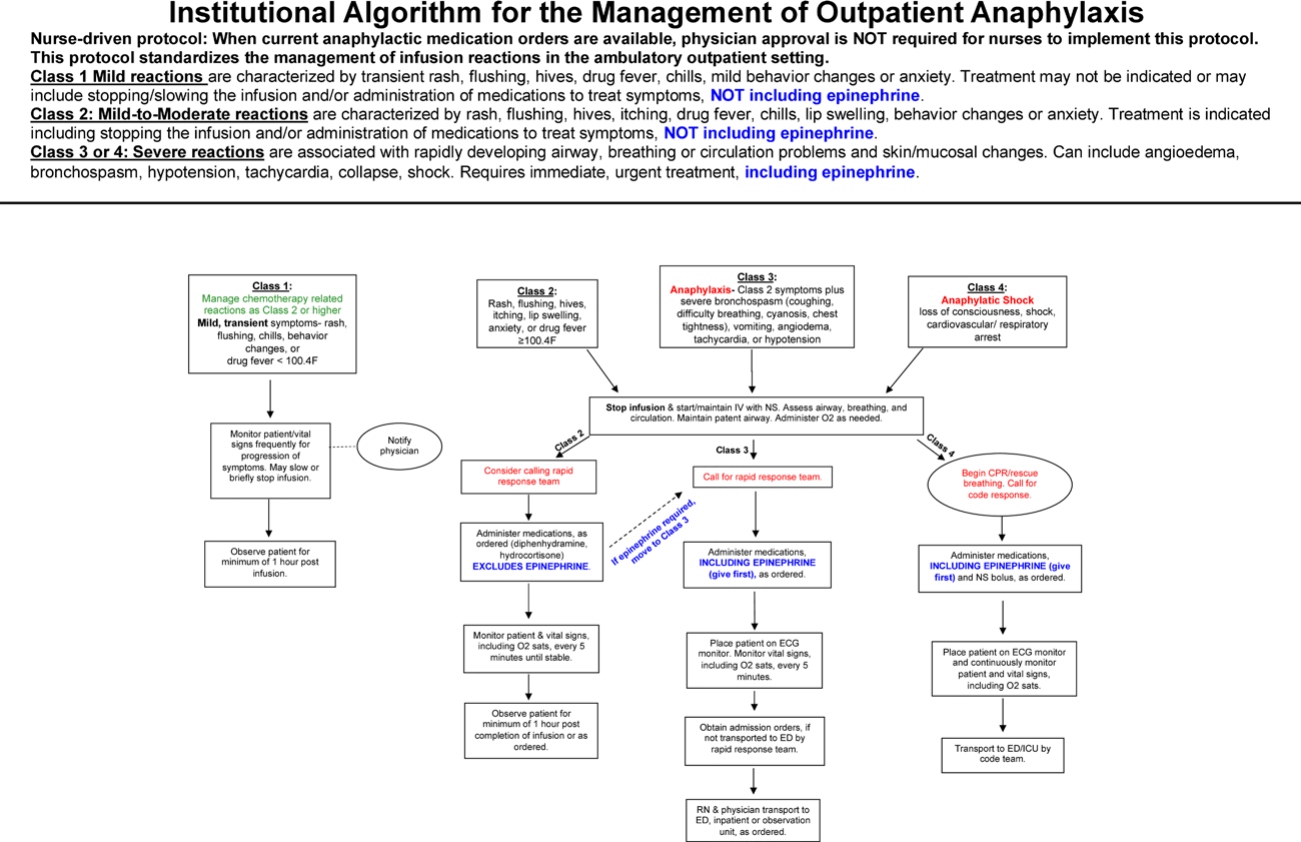
Institutional algorithm for the management of outpatient anaphylaxis. Algorithm detailing guidance for class 1–4 reactions to outpatient medications and infusions. The protocol is nurse-driven—when current anaphylactic medication orders are available, physician approval is not required for nurses to implement this protocol. This protocol standardizes the management of reactions in the ambulatory outpatient setting. ECG, electrocardiogram; ED, emergency department; ICU, intensive care unit; NS, normal saline; O_2_, oxygen; RN, registered nurse.

The AWG included the following team leaders: nurses, pharmacists, quality/safety officers, clinical informatics staff, key administrative leaders, and pediatric subspecialty physicians (eg, allergy/immunology, critical care, nephrology, and hematology/oncology). The AWG met monthly for over 2 years. They collected and reviewed anaphylaxis guidelines/order sets (eg, paper templates, electronic medical record ordering modules, pharmacy workflows, etc.) used across the institution and compared them with the WAO criteria for anaphylaxis.^1^ The physicians agreed on utilizing an evidence-based anaphylaxis algorithm that was easy to recognize and follow (Fig. 1). Nurse leaders educated nursing teams on anaphylaxis and provided updates on the anaphylaxis algorithm and EMR order set. Nurse educators trained nursing staff on properly recognizing and managing anaphylaxis to avoid treatment delay. Pharmacists helped implement the EMR order set and provided a cost-benefit analysis of epinephrine autoinjectors. EMR Information Technology developers worked to implement the Smart Set across all outpatient settings. There was some apprehension from the pharmacy department regarding the feasibility of implementing the created algorithm. To overcome this barrier, the AWG met with hospital administrators and executives to discuss incorporating the algorithm into the EMR and secure resources for programming.

The team dedicated 1 year to designing and implementing the goals of AWG. This work identified the causal factors contributing to potential patient safety events and/or non-standard-of-care practices. The AWG placed particular emphasis on the definition of a systemic allergic reaction requiring epinephrine and the appropriate dose and route for administering epinephrine in a given clinical situation. From this consensus evidence-based practice guideline, education materials were developed and distributed to all medical personnel across disciplines for phased implementation. Once implemented, the team dedicated an additional year to evaluating the safety of the protocol and implementation concerns. This protocol was implemented across 3 tertiary hospital outpatient settings, which also needed site-specific leadership approval and education.

## RESULTS

**Fig. 2.**
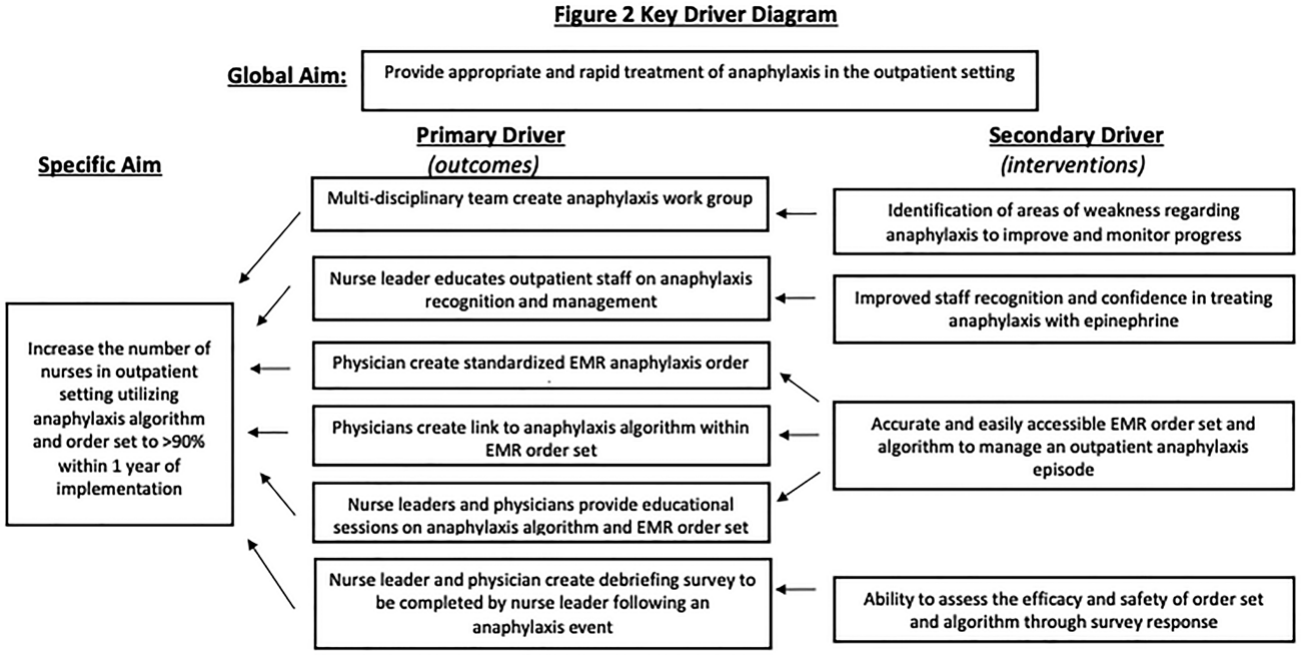
Key driver diagram. This diagram details the interventions and key drivers that contribute to helping achieve our global aim of providing appropriate and rapid treatment of anaphylaxis to medications in the outpatient setting.

Over 2 years, the A/I division identified that our hospital was using inappropriate routes and dosages of epinephrine for anaphylaxis in both the inpatient and outpatient settings across our integrated delivery system. As part of its assessments and investigations, the AWG identified 3 primary causal factors: (1) reluctance by medical staff to use epinephrine; (2) unclear indications for using IV versus IM epinephrine; and (3) systemic barriers to accessing necessary medications required to treat anaphylaxis. Through recognizing causal factors, key drivers can be identified and inserted into a key driver diagram (Fig. 2) that details our interventions. The AWG goal is to provide appropriate and rapid treatment of anaphylaxis in the outpatient setting. In addition, we aim to increase the number of nurses in the outpatient setting utilizing the anaphylaxis algorithm and order set to 90% within 1 year.

Nursing staff typically rely on physicians to diagnose anaphylaxis, which can delay necessary care. Thus, the AWG created an educational program and order set with the appropriate medications to help expedite proper care. The following concrete changes were made to the institutional anaphylaxis protocols across the integrated delivery system. First, the AWG reviewed the WAO Systemic Allergic Reaction Guidelines and revised the anaphylaxis protocol. Although WAO criteria were validated for outpatient immunotherapy infusions, this grading system may also be applied to any setting where systemic allergic reactions may occur. The AWG determined that a class 3 reaction or greater would require using IM epinephrine (Fig. 1).

Second, as IM epinephrine at the 1:1,000 concentration is the appropriate treatment for anaphylaxis, the AWG changed the anaphylaxis order set in the electronic medical record to include an IM epinephrine autoinjector option at 0.15 mg (EpiPen Jr) or 0.3 mg (EpiPen). Weight limits were indicated for each dose. In addition, EpiPen and EpiPen Jr were placed in all crash carts in the outpatient setting for quick and easy accessibility in urgent clinical situations. Finally, this information prompted the creation of teaching materials in the form of slide presentations. AWG educators distributed and explained this information to medical staff in the outpatient setting and encouraged medical staff to take the initiative in recognizing and promptly treating anaphylaxis.

**Fig. 3.**
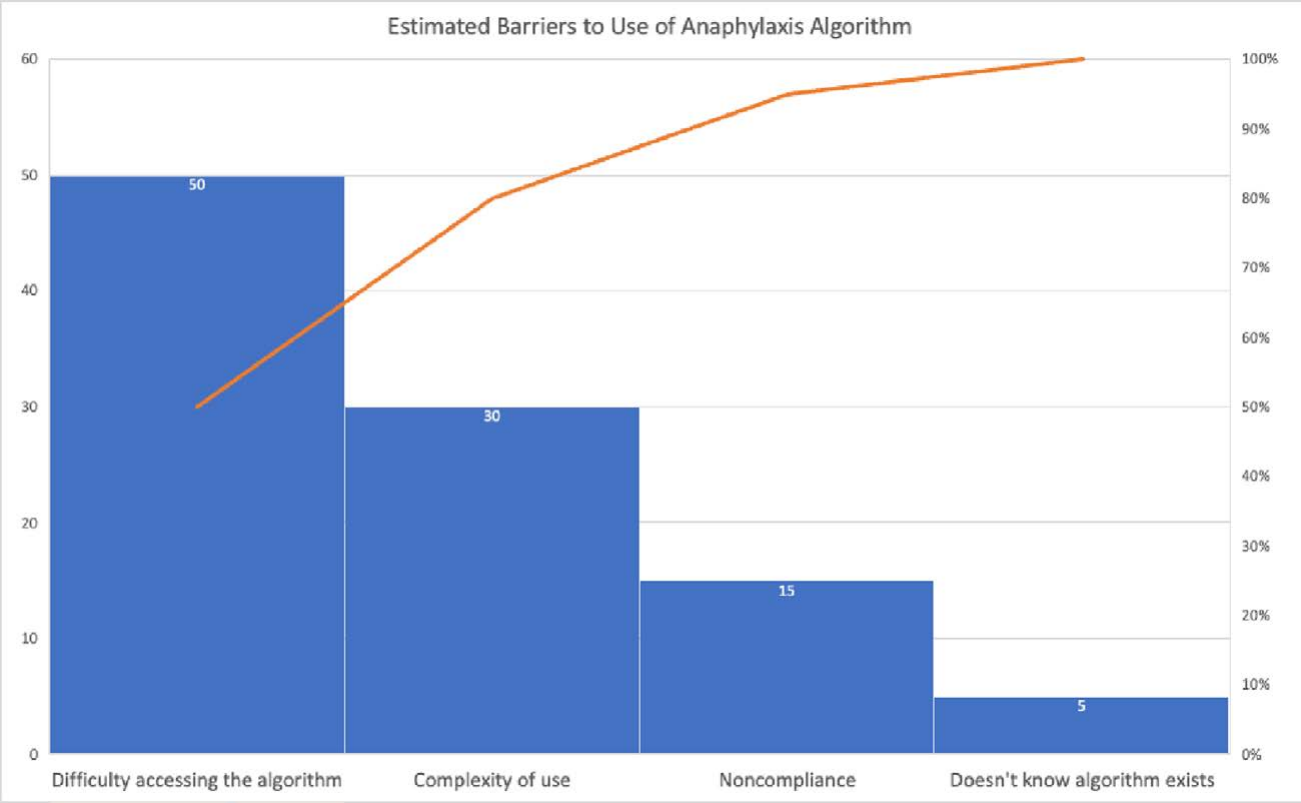
Pareto chart. This diagram provides an estimated breakdown of possible barriers to the implementation of our anaphylaxis algorithm.

By addressing the causal factors mentioned previously, the goal was to improve the following concrete outcomes: (1) the percentage of anaphylaxis episodes in which the correct dose and route of epinephrine administration were used; (2) the time between the onset of anaphylaxis and epinephrine administration; (3) the percentage of anaphylaxis episodes utilizing the anaphylaxis EMR order set and algorithm; and (4) the person ordering and administering epinephrine. These measures will help us gauge the outcome of proper recognition of anaphylaxis and increase nursing confidence to treat anaphylaxis independently. Additionally, with subjective feedback from the AWG, a Pareto chart, as seen in Figure 3, estimates the potential barriers to success, including (1) lack of knowledge regarding the existence of the algorithm; (2) difficulty accessing the algorithm; (3) algorithm noncompliance; and (4) complexity of using the algorithm.

## DISCUSSION

Although identifying signs/symptoms and indications for using epinephrine seem obvious on paper, in evolving clinical situations, especially with pediatric patients, signs/symptoms may present less clearly. For example, young patients may have difficulty articulating throat swelling or changes in vital signs, such as tachypnea. Clinicians may dismiss these symptoms in an anxious child. As such, there may be hesitancy from the medical staff in immediately using emergent life-saving medications, such as IM epinephrine (1:1,000). Patients may require admission to the intensive care unit after systemic drug reactions if improperly treated with only antihistamines, corticosteroids, and/or the 1:10,000 strength of epinephrine. Proactive education and ongoing scheduled training will help medical staff better differentiate when epinephrine is required for systemic allergic reactions or anaphylaxis.

Previously at our institution, confusion about when to use IM versus IV epinephrine stemmed from more frequent use of IV epinephrine, which was available in the emergency crash carts in the clinical setting, as well as multiple options being available to health care providers in the electronic medical record anaphylaxis order sets. In an urgent situation, clinicians should clearly and proactively identify the optimal medical treatment so that the correct treatment is given and not delayed. IM epinephrine should be indicated as the treatment of choice in every case of anaphylaxis. In addition, staff should be comfortable with the use of IM epinephrine devices. Videos and demonstrations with epinephrine autoinjector “trainers” are useful tools to help medical providers master the proper administration of IM epinephrine.

Finally, medical teams are well-versed and trained to use epinephrine in cases of cardiac arrest. However, it requires a different route of administration, dose, and concentration [IV epinephrine, 0.01 mg/kg (1:10,000)] than that required for anaphylaxis [IM epinephrine, 0.01 mg/kg (1:1,000)]. Frequent “code blue” simulations train medical teams to obtain IV epinephrine from a code cart and deliver it during these situations. However, scenarios for anaphylaxis are not commonly practiced in simulation sessions. Furthermore, although epinephrine autoinjectors are readily available in crash carts in some clinical settings (eg, A/I Clinics), they are not available in every setting.

Implementing anaphylaxis-related education and algorithms encourages staff to identify and treat anaphylaxis quickly. However, we understand that there will be barriers to using an algorithm. An outpatient nurse leader will complete a survey following an anaphylaxis event to address these barriers. This survey will ask if the algorithm was used, identify any barriers to its use, the dose and route of administration of epinephrine used, the time between symptom onset and administration of epinephrine, and who ordered and administered epinephrine. The AWG will collect and analyze these surveys to identify areas of improvement to the algorithm or evaluate if and when further educational sessions are needed. A limitation of our current work is the lack of robust outcome data to report, which we aim to build on in future works.

The use of quality improvement initiatives surrounding anaphylaxis has been detailed previously in the literature. For example, Kraft et al^11^ implemented standardized and documented care during anaphylaxis events in an A/I clinic and assisted in patient transfer to the emergency department. In addition, there have been multiple published studies^12,13^ related to decreasing hospitalization rates after anaphylaxis. These reports have focused on education on proper initial and adjunct treatment of anaphylaxis, EMR order set creation, and discharge goals and instructions. Our protocol also focused on provider education and implementing an EMR order set. However, this is the first published report highlighting some specific components of anaphylaxis education. We focused our initiative on outpatient drug reactions, so knowledge of non-IgE-mediated infusion reactions is also important. We also wanted to empower nursing staff to accurately identify and treat anaphylaxis with the correct medication without waiting for physician orders. This focus should help decrease delays in care and improve treatment outcomes.

## CONCLUSIONS

A near-miss patient safety event involving a delay in administering epinephrine for anaphylaxis led to creation of a comprehensive anaphylaxis work group, analysis of causal factors, and implementation of new anaphylaxis guidelines. The AWG created educational materials and distributed them to the outpatient setting. In addition, they substantially changed the EMR order set and added IM epinephrine autoinjectors to outpatient crash carts at our center. The next steps will include an analysis of the effects of these changes and an investigation of inpatient anaphylaxis protocols by the anaphylaxis work group to identify areas of improvement.

As novel treatments are being created and used in various clinical settings, systemic allergic reactions may also become more prevalent. Therefore, properly identifying and treating anaphylaxis is imperative to appropriate patient care. Using proper anaphylaxis guidelines and encouraging all medical staff to recognize and treat anaphylaxis, we can safely improve our patients’ safety and quality of care.

## DISCLOSURE

S. Anvari receives grant funding from the NIAID/NIH and DBV Technologies. M. D. Shah is currently employed by Genentech, Inc. (a member of the Roche Group). L. Forbes-Satter receives grant funding from the NIAID/NIH and NCATS/NIH. The other authors have no financial interest to declare.
